# Plant-Mediated Synthesis of Silver Nanoparticles and Their Stabilization by Wet Stirred Media Milling

**DOI:** 10.1186/s11671-017-1860-z

**Published:** 2017-02-01

**Authors:** Matej Baláž, Ľudmila Balážová, Nina Daneu, Erika Dutková, Miriama Balážová, Zdenka Bujňáková, Yaroslav Shpotyuk

**Affiliations:** 1 Department of Mechanochemistry, Institute of Geotechnics, Slovak Academy of Sciences, Watsonova 45, Košice, 04001 Slovakia; 2 Department of Pharmacognosy and Botany, University of Veterinary Medicine and Pharmacy, Komenského 73, Košice, 04181 Slovakia; 3 Department of Nanostructured Materials, Jožef Stefan Institute, Jamova cesta 39, Ljubljana, 1000 Slovenia; 4 Ivan Franko National University of Lviv, Tarnavskogo 107, Lviv, 79017 Ukraine; 5 CITNSEK, Faculty of Mathematics and Natural Sciences, University of Rzeszow, Pigonia 1, Rzeszow, 35-958 Poland

**Keywords:** Silver NPs, Plant-mediated synthesis, Origanum vulgare L., Mechanochemistry, Polyvinylpyrrolidone

## Abstract

Within this study, a stable nanosuspension of silver nanoparticles (Ag NPs) was prepared using a two-step synthesis and stabilization approach. The Ag NPs were synthesized from a silver nitrate solution using the *Origanum vulgare* L. plant extract as the reducing agent. The formation of nanoparticles was finished upon 15 min, and subsequently, stabilization by polyvinylpyrrolidone (PVP) using wet stirred media milling was applied. UV-Vis spectra have shown a maximum at 445 nm, corresponding to the formation of spherical Ag NPs. Infrared spectroscopy was used to examine the interaction between Ag NPs and the capping agents. TEM study has shown the formation of Ag NPs with two different average sizes (38 ± 10 nm and 7 ± 3 nm) after the plant-mediated synthesis, both randomly distributed within the organic matrix. During milling in PVP, the clusters of Ag NPs were destroyed, the Ag NPs were fractionized and embedded in PVP. The nanosuspensions of PVP-capped Ag NPs were stable for more than 26 weeks, whereas for the non-stabilized nanosuspensions, only short-term stability for about 1 week was documented.

## Background

Silver nanoparticles are in the spotlight of researchers these days [1, 2]. Their multidisciplinary application is generally known [3], although their utilization as antibacterial agents is probably the most widely studied [4, 5].

There are various synthetic methods to obtain silver NPs [6], e.g., laser ablation, gamma irradiation, electron irradiation, chemical reduction, photochemical methods, microwave processing, and biological synthetic methods [7]. Regarding the silver precursors for the Ag NPs synthesis, most often silver nitrate is used, as it is inexpensive and well accessible. A reducing agent has to be applied in order to transform silver from ionic to elemental form.

In the so-called green approach, the reduction procedure is performed by a natural-based material, most commonly a plant extract containing substances with the reducing properties. Various plants were applied for the synthesis of Ag NPs, and this topic has been the focus of numerous review papers in the last years [8–10]. Among rich plethora of the plants, also *Origanum vulgare* L. (in further text referred to as *O. vulgare* L.) was used for the synthesis of Ag NPs [11]. This plant is quite common and it contains substances with health-beneficial properties [12, 13], and it has one of the strongest known antioxidant activity among culinary herbs [14].

In most cases, silver nanoparticles need to be functionalized by various capping agents prior to their application. The capping agents can be also the substances responsible for the reduction of silver, or some other components present in the plants [8]. Their main function is to increase the stability of NPs in a solution, which is the key factor when considering the suitability of the capping agent for the nanosuspension preparation [15, 16]. Capping agents can also affect the shape of NPs [17–19]. Polyvinylpyrrolidone (PVP) belongs among the most widely used capping agents, as it is non-toxic [20] and can serve various beneficial purposes during or after the synthesis of nanoparticles. It can be applied as surface stabilizer, growth modifier, nanoparticle dispersant, or reducing agent [21]. Moreover, its beneficial effect on the antimicrobial properties of Ag NPs in combination with antibiotics was reported in literature [22, 23].

Within this study, Ag nanoparticles were prepared using the *O. vulgare* L. water extract according to a slightly modified procedure proposed by Sankar et al. [11]. The prepared Ag NPs were subsequently stabilized in PVP by wet stirred media milling, and we have compared the characteristics of the PVP-stabilized Ag NPs with the non-stabilized ones.

## Methods

### Materials

Silver nitrate (AgNO_3_, 99.9%, Mikrochem, Slovakia) and polyvinylypyrrolidone (PVP, Sigma-Aldrich, Great Britain) were used as chemicals without further purification. *O. vulgare* L. plants were collected in summer from the meadow in University of Veterinary Medicine and Pharmacy campus in Košice, Slovakia.

### Preparation of *O. vulgare* L. Water Extract (ORE)

Whole plants of the *O. vulgare* L. plants (flowers, leaves, and stems) were dried up to constant weight in dark at room temperature. The dried above-ground parts of plants were powdered to fine particles by a mixer. *O. vulgare* L. extract was prepared by suspending 10 g of the dry powder into 100 mL of distilled water. The mixture was heated at 60 °C for 10 min, and after cooling down, the solid residues were removed and the filtrate was used for the synthesis of nanoparticles.

### Plant-Mediated Synthesis of Ag NPs (Ag/ORE)

Silver nitrate water solution with the concentration 1 mM was prepared just before application under dark conditions to prevent its decomposition. During continuous shaking, 90 mL of AgNO_3_ solution was heated up to 80 °C and 10 mL of *O. vulgare* L. water extract was slowly added to the hot solution and incubated in the temperature range 75–85 °C using a water bath for 15 min. The formation of Ag NPs was observed by transformation from the light brownish yellow to the dark brownish red color monitored by UV-Vis spectroscopy (see Preparation section in Results and Discussion).

### Stabilization of Ag NPs in PVP (Ag/ORE/PVP)

The stabilization by wet stirred media milling was realized in a Minicer laboratory stirring media mill (Netzsch, Germany). 1.5 g of PVP was dissolved in 300 mL of the Ag/ORE nanosuspension, thus forming a 0.5% water solution. The used milling speed was 2000 rpm, and as milling media, yttrium-stabilized ZrO_2_ milling balls with the diameter 0.6 mm were used. The milling time was 60 min. After milling, the nanosuspensions were stored in refrigerator at 4 °C.

### Characterization Methods

The progress of Ag NPs formation was monitored by recording Vis spectra every minute using the Gary 60 UV-Vis spectrophotometer (Agilent Technology, Malaysia). For characterization of the final samples, UV-Vis spectrophotometer Helios Gamma (Thermo Electron Corporation, Great Britain) working in the range 200–800 nm was used.

Particle size distribution of the nanosuspensions was measured by a photon cross-correlation spectroscopy (PCCS) using a Nanophox particle size analyzer (Sympatec, Germany). A portion of each nanosuspension was diluted to achieve a suitable concentration for the measurement. This analysis was performed using a dispersant with the refractive index of 1.33. The measurements were repeated three times for each sample.

Room-temperature photoluminiscence (PL) spectra were acquired at the right angle on a photon counting spectrofluorometer PC1 (ISS, USA) with an excitation wavelength of 330 nm. A 300-W xenon lamp was used as the excitation source. The emission was collected in a 25-cm monochromator with a resolution of 0.1 nm. The monochromator was equipped with a photomultiplier. The emission spectra were obtained out in the range of 350 to 650 nm. The excitation and emission slit widths were kept at 2 and 1 nm, respectively.

Zeta potential (ZP) values were measured using a Zetasizer Nano ZS (Malvern, Great Britain). The ZP values were obtained by applying the Helmholtz-Smoluchowski equation built into Malvern zetasizer software. ZP was measured in the original dispersion medium, and the measurements were repeated three times for each sample.

FT-IR spectra were recorded using a Tensor 29 infrared spectrometer (Bruker, Germany) using the ATR method. In the case of solid samples (AgNO_3_ and PVP), directly, the chemicals were analyzed. In the case of liquid solutions (ORE) or suspensions (Ag/ORE, Ag/ORE/PVP), few drops were transferred onto a watchglass and dried at 70 °C prior to measurement. Then, the dry substance was scratched from the watchglass and the resulting powder was analyzed.

The size, shape, and chemical composition of the nanoparticles and the capping agents were analyzed by transmission electron microscopy (TEM). Nanosuspensions were ultrasonically homogenized for 5 min. A droplet of the suspension was applied onto a lacey carbon-coated nickel grid and dried. Prior to the TEM analyses, the samples were carbon-coated to prevent charging under the electron beam. TEM analyses were performed using a 200-kV microscope JEM 2100 (JEOL, Japan) with LaB_6_ electron source and equipped with energy dispersive X-ray spectrometer (EDS) for chemical analyses.

## Results and Discussion

### Preparation

#### Plant-Mediated Synthesis of Ag NPs (Ag/ORE)

**Fig. 1:**
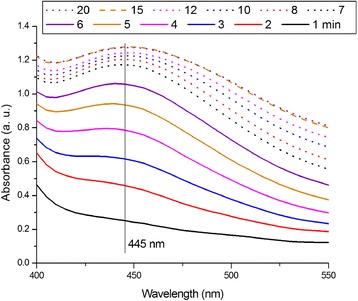
Vis spectra taken during the synthesis of Ag NPs, reaction time in minutes is provided in the *legend above the graph*

The course of the Ag nanoparticle synthesis was observed by the measurement of the Vis spectra, which were taken every minute during the synthesis (Fig. 1).

The Vis spectra show that almost immediately after the mixing of *O. vulgare* L. water extract (in further text abbreviated as ORE) and AgNO_3_ solution, the absorbance at 445 nm appears. With prolonged synthesis time, the absorbance increases and develops into the peak maximum. This peak indicates spherical Ag NPs with size in the range of tens of nanometer [24]. The localization of the peak maximum is influenced by both, the size and the shape of the Ag NPs [24–26]. The time-lapse Vis spectra provide information about kinetics of the nanoparticle formation reaction. The reaction proceeds more quickly within the first 7 min while later, kinetics of the reaction is slowed down as the differences between spectra become smaller. After 15 min, the absorbance does not increase anymore, indicating that the reaction is completed. The time of 15 min is in accordance with reports by other authors, where plant-mediated synthesis was realized [27–29].

#### Stabilization of the Ag NPs by Wet Stirred Media Milling in PVP (Ag/ORE/PVP)

**Fig. 2:**
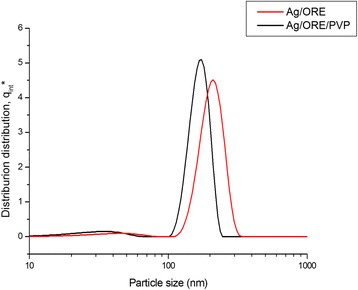
Particle size distribution before (Ag/ORE) and after (Ag/ORE/PVP) stabilization

In order to stabilize the Ag NPs prepared by the plant-mediated synthesis, wet milling in 0.5% water solution of PVP capping agent was applied. The effect of milling on the particle size distribution can be demonstrated by comparing the distribution curves for Ag/ORE and Ag/ORE/PVP samples (Fig. 2).

Usually, when PVP is involved as a capping agent in the synthesis of Ag NPs, it is added as one of the reagents in a one-step reaction [19]; however, in our case, it was added in the second step, after the completion of the plant-mediated synthesis. The particle size of Ag NPs was measured after each synthesis step (Fig. 2). The size of the Ag NPs of Ag/ORE sample appears to be bimodal. The main fraction of particles is in the range between 110 and 330 nm, with the absolute maximum located at 210 nm. The smaller fraction exhibits particle size between 15 and 80 nm. The average hydrodynamic particle diameter, x_50_, is 203 nm for this sample. Bimodal particle size distribution is observed also in the sample after 60 min of milling in PVP (Ag/ORE/PVP); however, the average size of both fractions decreased slightly. The absolute maximum for this sample is located at 174 nm, and the x_50_ value is 165 nm. In general, it can be concluded that milling brings about a slight decrease of the nanoparticle size and results in a narrower particle size distribution.

### Optical Properties

**Fig. 3:**
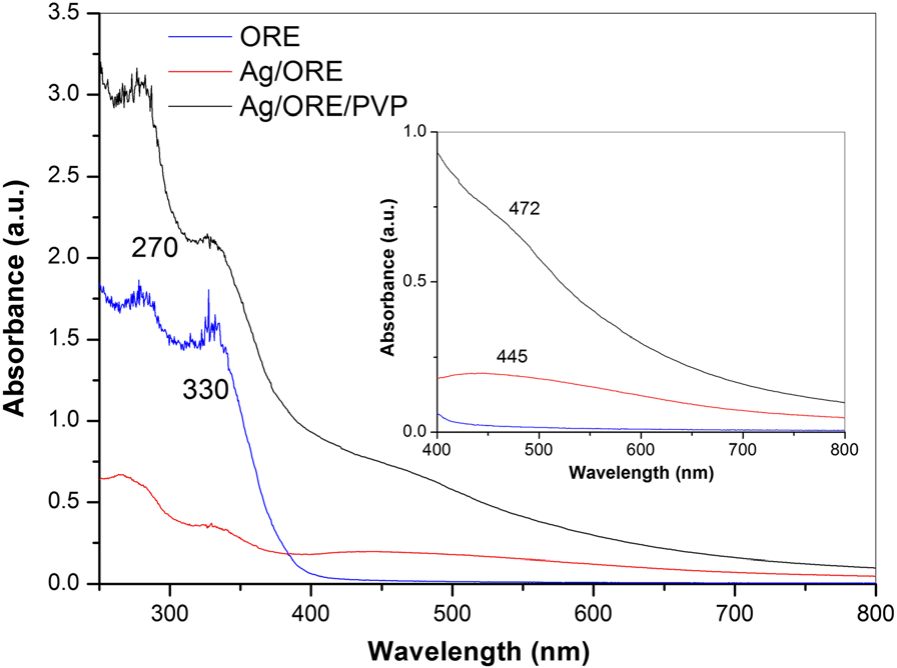
UV-Vis spectra of pure ORE, Ag/ORE, and Ag/ORE/PVP samples

UV-Vis spectroscopy is one of the basic tools for characterization of optical properties of Ag NPs [30]. Based on the UV-Vis spectra, properties of Ag NPs in terms of their size and shape can be determined. Results of the UV-Vis spectroscopy for the Ag/ORE and Ag/ORE/PVP samples and their comparison with the spectrum taken from ORE are shown in Fig. 3.

The UV-Vis measurements show that the pure ORE exhibits two peaks in the ultraviolet region centered at 270 and 330 nm, corresponding to 4.56 and 3.73 eV, respectively. In the visible region (inset of Fig. 3), no peak is present. In the case of Ag/ORE sample, additional peak at 445 nm (2.77 eV) can be observed, which it is in accordance with earlier published results [11, 31] and can be related to the formation of spherical-like Ag NPs [32], as mentioned earlier. UV-Vis spectrum of the Ag/ORE/PVP sample displays a peak at 472 nm (2.61 eV). This peak is red-shifted compared to the Ag/ORE sample, which could imply a change in the Ag particle size or shape. It is known that addition of PVP can dramatically change the morphology of Ag nanomaterials, e.g., from spherical shape to nanowires, especially when PVP is used directly as a capping agent during the synthesis. One of the most important factors influencing the formation of nanoparticles with different morphology is the PVP to AgNO_3_ ratio [19, 33]. In the present case, PVP was applied after the Ag NPs formation; therefore, its effect on the morphology of the Ag nanoparticles is expected to be different compared to the addition during the Ag NPs synthesis. The effect of PVP on the morphology of the Ag NPs will be revealed by TEM.

**Fig. 4:**
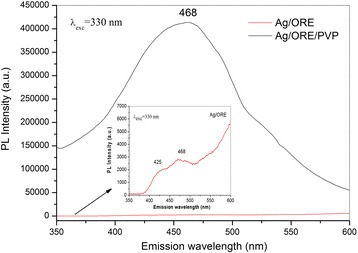
PL spectra of Ag/ORE and Ag/ORE/PVP

Optical properties of the Ag NPs before and after stabilization with PVP were analyzed by PL spectroscopy (Fig. 4), as this method is also suitable for the analysis of metallic NPs [34].

PL spectra clearly show that the prepared Ag NPs exhibit photoluminescence due to excitation of electrons from occupied d bands into states above the Fermi level. Subsequent electron–phonon and hole–phonon scattering process leads to an energy loss and finally photoluminescent radiative recombination of an electron from an occupied sp band with the hole [32]. In the present study, the PL spectrum of Ag/ORE measured with the excitation wavelength 330 nm in the region 350–650 nm revealed the very weak broad emission peaks at 425 nm (2.89 eV) and 468 nm (2.63 eV). The observed peak at 468 nm is in accordance with the peak corresponding to Ag nanoparticles prepared by chemical citrate reduction noticed in paper [31]. In the case of Ag/ORE/PVP sample, a broad emission peak located at 468 nm with very high PL intensity is observed indicating that the introduction of PVP into the system enhances the photoluminescence emission intensity.

### Interaction of Ag NPs with the Capping Agents

#### Infrared Spectra

**Fig. 5:**
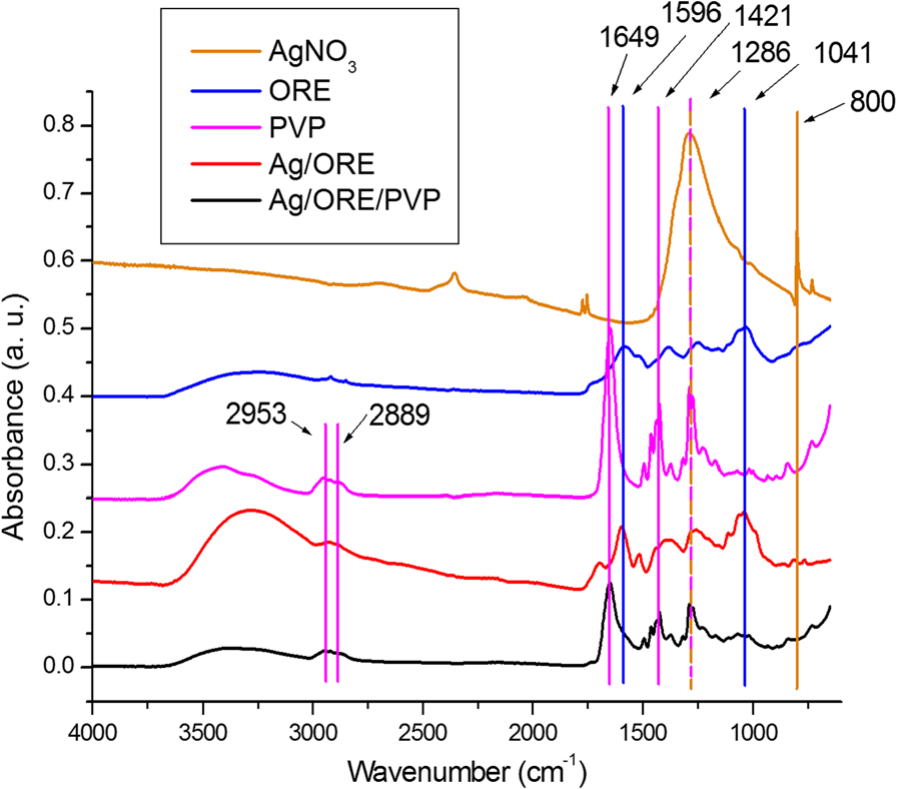
Infrared spectra of pure silver nitrate, dried ORE, PVP, Ag/ORE, and Ag/ORE/PVP samples

In order to investigate the potential formation of bonds in the present system, the FT-IR spectra were recorded (Fig. 5).

FT-IR spectra of AgNO_3_, dried ORE and PVP were recorded and compared to the spectra of the Ag/ORE and Ag/ORE/PVP samples. The spectrum of silver nitrate shows a wide peak at 1286 cm^−1^, which coincides with the vibration from PVP, so from this peak, it cannot be claimed for sure that there is no silver nitrate present in the Ag/ORE/PVP sample. However, the absence of the peak at 800 cm^−1^ in the Ag/ORE, and subsequently Ag/ORE/PVP samples, provides definite proof of successful washing out of AgNO_3_. The spectrum from the ORE contains many peaks since it is a complex system containing a huge amount of different substances [35]. Some suggestions for the presence of amide, nitrile, and aromatic groups can be found in [11]. Also, the broad peak at 3267 cm^−1^ should be associated with the presence of –OH group. Some peaks of the ORE (e.g., the 1596 and 1041 cm^−1^) show a slight shift to lower wavenumbers in the case of Ag/ORE sample. This shift is in accordance with [36] and indicates possible successful interaction between the formed Ag NPs and some components of ORE.

Upon addition of PVP to the Ag/ORE sample, only the peaks belonging to the polymer could be registered in the Ag/ORE/PVP sample. On the contrary to other studies [33, 37], we did not observe any change in the positions of the peaks in the infrared spectra after the interaction between PVP and our Ag/ORE system. The peaks corresponding to C=O vibration at 1649 cm^−1^, C–N vibration at 1286 cm^−1^, nor the CH_2_ stretching vibrations at 2953 and 2889 cm^−1^, did not shift at all. The same is valid also for the peak at 1421 cm^−1^. If interactions of chemical character would occur in the sample, changes in the intensity (or position) of some peaks should be visible. Namely, PVP possesses two places for interaction, concretely oxygen and nitrogen atom [38], as was described also for the milled samples in [39]. Nevertheless, the stability of the system was significantly improved in the present case after the addition of PVP, as will be manifested later (see part Stability of the Prepared Ag NPs section).

A possible explanation for these observations could be that the PVP behaves like a matrix between the Ag/ORE individual systems, and its steric properties cause the particles not to agglomerate. This interaction can be possibly based only on some repulsion forces. Another explanation could be that only a small fraction of the used PVP molecules interact with the present system and the rest, being major, remains unaffected and shows in the spectrum after the drying. However, at least some changes should be observed if a chemical bond is formed. This needs to be studied in more detail in the future.

#### Zeta Potential Measurements

In this work, a two-stage procedure for the synthesis and stabilization of the Ag NPs was performed. ZP provides important information about the stability of the prepared Ag NPs and their possible interaction with ORE and/or PVP. The obtained results, together with the corresponding pH values, are presented in Table 1.

**Table 1:** Zeta potential and pH values for ORE, Ag/ORE, and Ag/ORE/PVP samples

Sample	Zeta potential (mV)	pH
ORE	–	6.01
Ag/ORE	−18.4	3.67
Ag/ORE/PVP	−9.3	6.00

Pure extract exhibited pH 6.01, which decreased significantly after the production of Ag NPs (Ag/ORE), thus documenting a change in the charge distribution. The recorded ZP was −18.4, which is lower than −26 mV reported in [11]. This difference could result from the fact that different substances are extracted when both flowers and leaves from *O. vulgare* L. are used. Upon the addition of PVP, the ZP has decreased to −9.3 mV. This decrease is due to steric stabilization provided by PVP [40] and was also noticed in [39], where the stabilization of nanoparticles with PVP was also performed by the milling process. After milling in PVP, the pH increased again to a more neutral value, as PVP exhibits slightly alkaline pH.

### TEM Analysis of the Ag NPs

**Fig. 6:**
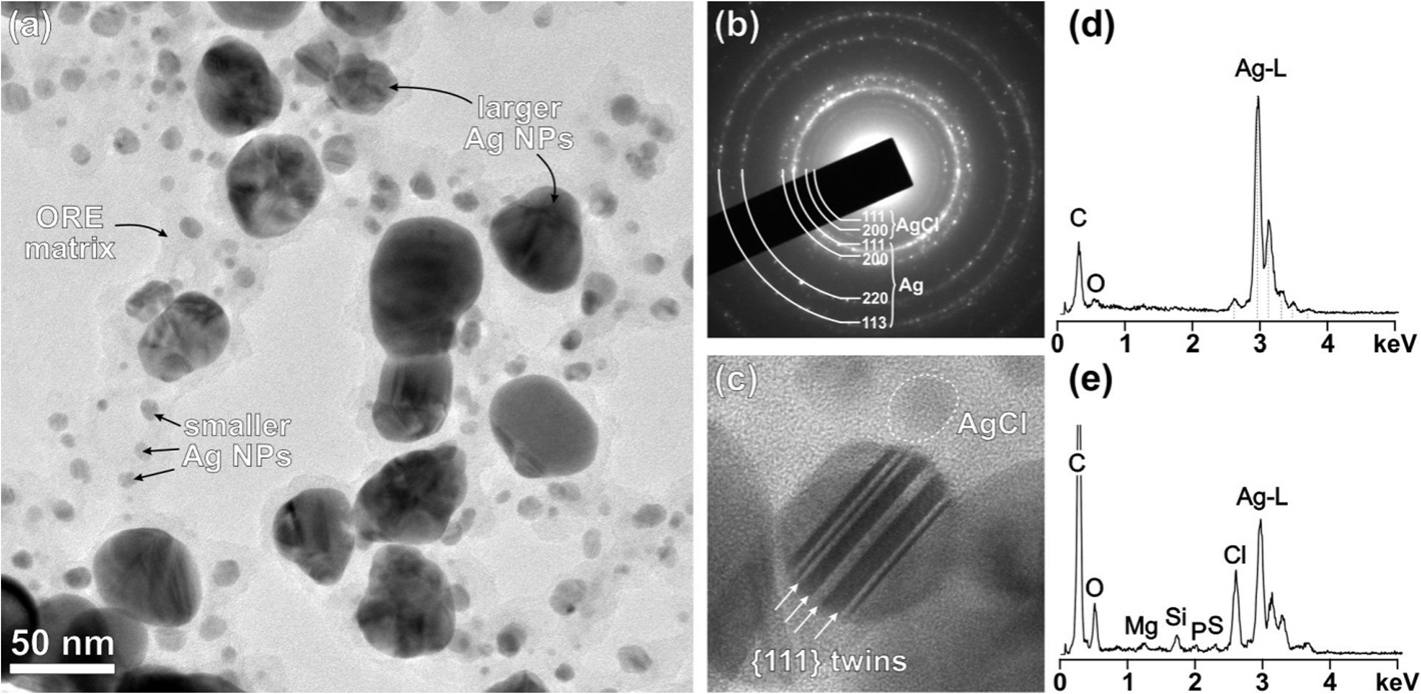
TEM analysis of the Ag/ORE sample. **a** Low-magnification image shows the bimodal particle size distribution. **b** SAD pattern can be indexed by Ag and some AgCl phases. **c** Typical for the larger Ag NPs is the presence of parallel twins; a small particle of AgCl displaying weaker contrast due to the lower average density is *encircled*. **d** EDS spectrum from one larger Ag NP and **e** EDS spectrum taken from several small Ag NPs embedded in ORE matrix

The size and morphology of the Ag NPs and the presence and distribution of the capping agents in the samples was studied by TEM. Figure 6a shows a typical image of the Ag NPS after the plant-mediated synthesis (Ag/ORE sample). In this sample, the Ag NPs are embedded in amorphous matrix, originating from the ORE. The Ag NPs have two distinctly different particle sizes (bimodal particle size distribution); the average diameter of the larger particles is 38 ± 10 nm while the smaller fraction has a mean diameter of 7 ± 3 nm. This observation is not in full agreement with the PCCS (see Stabilization of the Ag NPs by Wet Stirred Media Milling in PVP (Ag/ORE/PVP) section, Fig. 2) where bimodal particle size distribution with the larger fraction around 210 nm and finer fraction between 15 and 80 nm was determined. Since no particles with diameter larger than ~50 nm were observed in the TEM, the results of the PCCS measurement may be a result of agglomeration of NPs into larger clusters. Moreover, in the case of PCCS, the sample during measurement is in liquid form. While the smallest NPs (those below 10 nm) are almost spherical, the larger NPs exhibit typical pseudooctahedral morphologies (octahedra with rounded edges), where flat sections of the facets run parallel with the {111} planes. This morphology is close to spherical (isometric), as indicated by the Vis spectra measurements (maximum at 445 nm; see Fig. 1).

Crystallinity of the Ag NPs was inspected by selected-area electron diffraction (SAD). A typical SAD pattern recorded with the largest aperture is shown in Fig. 6b. It is a ring-pattern typical for randomly oriented nanoparticles. Indexing of the rings revealed that besides Ag, also a small fraction of AgCl (halite-type structure) is present in the sample. Two characteristic peaks of the AgCl with larger *d*-values than any peak belonging to the Ag phase are reflections from {111} and {200} with *d*-values of 0.32 and 0.28 nm, respectively. All other diffraction rings belong to Ag with the copper-type structure. AgCl has much lower average atomic density compared to metallic Ag; therefore, the AgCl NPs have much weaker contrast in TEM. One of the AgCl NPs is shown in the Fig. 6c. This figure shows another typical feature of the larger Ag NPs, namely many of them show the presence of parallel twins. This type of twinning is not uncommon in Ag; however cyclic twins are more typical. The reason for the formation of parallel twins is not exactly known, it may lie in the specific nucleation and growth conditions within the matrix stemming from the *O. vulgare* L.-based reducing extract. Similar twins in Ag NPs were observed by Personick et al. [41], who synthesized Ag NPs with plasmon-mediated synthesis and showed that excitation wavelength is an important parameter for controlling particle twinning. They observed the formation of planar twins in Ag NPs only under 500 nm excitation while under 400 nm, multiple twins were not formed.

Chemical composition of the nanoparticles and the matrix were further analyzed by EDS. A spectrum taken from one larger Ag NP contains mainly Ag, the presence of a small amount of C may be from the carbon foil and coating, while O from matrix surrounding the nanoparticles (Fig. 6d). The spectrum from a larger area containing several smaller Ag NPs embedded in the matrix, on the other hand, shows that besides Ag from the smaller NPs, other elements are present in this area like quite a high amount of Cl, distinctly more C and some Mg, Si, P, and S. All these elements most likely originate from the compounds present in the ORE matrix, which may have a complex chemical composition with many different groups as indicated from the FT-IR spectra. These compounds are responsible for the reduction of silver nitrate to Ag and also to its further reaction to the AgCl phase. In EDS, analyses of this sample N was not detected confirming efficient reduction of silver nitrate by the oregano extract, as already suggested by FT-IR analyses (Fig. 5).

**Fig. 7:**
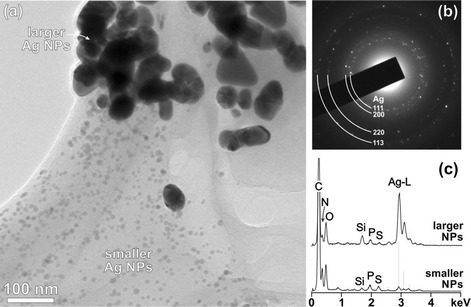
**a** TEM analysis of the Ag/ORE/PVP showing separation of the larger and smaller fraction of the Ag NPs. **b** SAD pattern from the larger Ag NPs. **c** EDS analysis of the areas containing larger and smaller Ag NPs

In addition to the Ag NPs prepared by plant-mediated synthesis (Ag/ORE), the sample after milling in PVP (Ag/ORE/PVP) was also analyzed under TEM and the results are shown in Fig. 7.

Low-magnification TEM image of the Ag/ORE/PVP sample reveals that during the milling process, clusters of the ORE matrix with larger and smaller Ag NPs that formed after the plant-mediated synthesis (Fig. 6a) were destroyed. The larger Ag NPs are occasionally found as individual particles scattered around the TEM grid or embedded in the PVP matrix, sometimes together with some remnants of the ORE matrix. It is interesting to note that both fractions of the Ag NPs separated during the milling process and were found concentrated in different parts of the sample (Fig. 7a). The smaller Ag NPs are always embedded in the PVP matrix. In this sample, diffraction analyses did not show the presence of any phase other than Ag (Fig. 7b). Also, the EDS analyses, taken either from the areas containing the large or small Ag NPs, did not show the presence of Cl (Fig. 7c), whereas other elements like Si, P, and S (related to the ORE extract) remained in the sample. This indicates leaching of Cl from the sample during milling in PVP. In both spectra, some N from the PVP matrix was detected.

### Stability of the Prepared Ag NPs

**Fig. 8:**
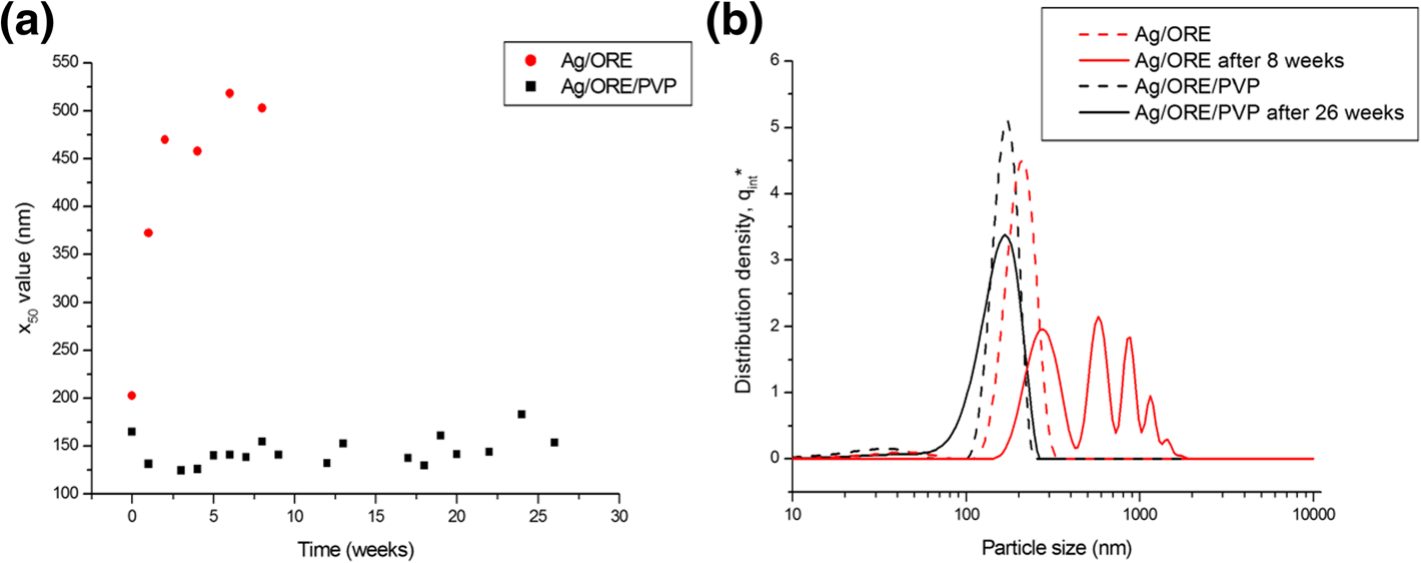
Stability of the Ag/ORE and Ag/ORE/PVP nanosuspensions. **a** Dependance of the mean particle size, x_50_, on the time of storage. **b** Particle size distribution in nanorange for the selected samples

The stability of the prepared nanosuspensions was tested every week after their preparation by the PCCS method. When the formation of the agglomerates, resulting in the polymodal distribution of NPs, was observed and the increase of the average hydrodynamic particle diameter (x_50_), the sample was considered non-stable. The values of this parameter for the two samples during weeks of storage are presented in Fig. 8a. For comparison, also the particle size distribution curves for the first and the last checked Ag/ORE and Ag/ORE/PVP samples are presented in Fig. 8b.

It can be clearly seen from the figure that the introduction of PVP brought about a significant improvement in the stability of the nanosuspensions, as the x_50_ value did not increase significantly until 26 weeks of storage at 4 °C. In the case of Ag/ORE sample, the significant increase in x_50_ value was observed even after one week of storage. These results document the successful stabilization by PVP and the suitability of this approach for the application needing long-term storage of the nanosuspensions.

## Conclusions

The successful synthesis of Ag nanoparticles using a green approach, namely the water extract of *O. vulgare* L. plant, was achieved. The formation of Ag nanoparticles was completed upon 15 min, as was documented by the Vis spectra. The UV-Vis spectroscopy has shown the absorption maximum at 445 nm, and this was red-shifted to 472 nm when applying PVP as a stabilization agent. The photoluminescence properties were also altered after the stabilization. Despite the fact that infrared spectroscopy did not show any chemical changes in the structure of PVP, its application dramatically enhanced the stability of the nanosuspension documented by the long-term monitoring of particle size distribution by PCCS. TEM analyses have shown the formation of smaller and larger nanoparticles, the former group possessing sizes around 7 nm and the latter one around 38 nm. These two groups of NPs were intermixed after the *Origanum*-mediated synthesis; however, after the stabilization with PVP, these regions were separated. The present study shows the possibility of an effective stabilization of the nanosuspension prepared by a green plant-mediated synthesis in the second step using a wet stirred media milling. The antibacterial activity of the prepared nanosuspensions will be studied in close future.

## Abbreviations

EDS: Energy dispersive X-ray spectrometer
ORE: *Origanum vulgare* L. water extract
PCCS: Photon cross-correlation spectroscopy
PL: Photoluminescence
PVP: Polyvinylpyrrolidone
SAD: Selected-area electron diffraction
TEM: Transmission electron microscopy
ZP: Zeta potential
